# Prevalence of and risk factors for methicillin-resistant *Staphylococcus aureus* colonization among human immunodeficient virus–infected outpatients in Taiwan: oral *Candida* colonization as a comparator

**DOI:** 10.1080/20002297.2017.1322446

**Published:** 2017-05-09

**Authors:** Chi-Jung Wu, Wen-Chien Ko, Mao-Wang Ho, Hsi-Hsun Lin, Yun-Liang Yang, Jiun-Nong Lin, I-Wen Huang, Hui-Ying Wang, Jui-Fen Lai, Yih-Ru Shiau, Li-Yun Hsieh, Hui-Ting Chen, Chih-Chao Lin, Wen-Li Chu, Hsiu-Jung Lo, Tsai-Ling Lauderdale

**Affiliations:** ^a^ National Institute of Infectious Diseases and Vaccinology, National Health Research Institutes, Zhunan, Taiwan; ^b^ Department of Internal Medicine, National Cheng Kung University Hospital, College of Medicine, National Cheng Kung University, Tainan, Taiwan; ^c^ Section of Infectious Diseases, Department of Internal Medicine, China Medical University Hospital, Taichung, Taiwan; ^d^ Division of Infectious Diseases, Department of Internal Medicine, E-Da Hospital, I-Shou University, Kaohsiung, Taiwan; ^e^ Institute of Molecular Medicine and Bioengineering, National Chiao Tung University, Hsinchu, Taiwan; ^f^ Department of Biological Science and Technology, National Chiao Tung University, Hsinchu, Taiwan; ^g^ School of Dentistry, China Medical University, Taichung, Taiwan

**Keywords:** HIV, *Staphylococcus aureus*, MRSA, *Candida*, oral colonization, HAART

## Abstract

Human immuodeficency virus (HIV)-infected patients receiving highly active antiretroviral therapy (HAART) and community-associated methicillin-resistant *Staphylococcus aureus* (CA-MRSA) have increased in recent years in Taiwan. This study was undertaken to determine the prevalence of and risk factors for nasal and oral *S. aureus* and MRSA colonization among contemporary HIV-infected populations. Clinical variables for *S. aureus* and MRSA colonization among HIV-infected outpatients from three hospitals were analyzed and compared with those for oral *Candida* colonization. Genetic characteristics of MRSA isolates were analyzed. A total of 714 patients were screened for nasal *S. aureus* colonization, and a subset of 457 patients were also screened for oral *S. aureus* colonization. Of all patients, 79.4% were receiving HAART, and their mean CD4 count was 472 cells/mm^3^. The colonization rates in the oral cavity, nasal cavity, and at either site were 18.8%, 31.7%, and 36.8%, respectively, for *S. aureus*, and 3.1%, 4.4%, and 5.5%, respectively, for MRSA. These rates were all much lower than the previously reported rate of oral *Candida* colonization (52.4%). By multivariate analysis, a suppressed viral load (<200 copies/mL) protected against oral *S. aureus*, MRSA, and *Candida* colonization, and recent use of antibacterial agents protected against oral and nasal *S. aureus* colonization. Recent incarceration increased the risk of nasal MRSA colonization, while recent hospitalization, tuberculosis, older age, and intravenous drug use increased the risk of oral *Candida* colonization. *Candida* spp. did not augment *S. aureus* or MRSA colonization in the oral cavity. Most of the 41 MRSA isolates recovered belonged to the SCC*mec* IV/*pvl*-negative (51.2%) and V_T_/*pvl*-positive (26.8%) ST59 local prevalent CA-MRSA clones. Distinct carriage rates demonstrated here suggested that mucosal immunity against colonization might differ in terms of microbes and sites. A decreased risk in oral carriage of MRSA and *Candida* might be a benefit of HAART.

## Introduction

*Staphylococcus aureus* and *Candida* species, both existing as common commensals colonizing human mucosal surfaces, are the leading opportunist pathogens causing human infections. In one study enrolling human immunodeficiency virus (HIV) patients with bacteremia attending the emergency department, *S. aureus* was found to be the most common (44.7%) causative pathogen [1]. Among *S. aureus*, methicillin-resistant *S. aureus* (MRSA) is most noteworthy because it is responsible for an increasing number of hospital- and community-acquired infections worldwide and because of its association with multidrug resistance [2]. Studies have revealed synergistic interactions between *S. aureus* and *Candida albicans* in dual-species biofilms, which is consistent with clinical findings of frequent co-isolation of both species from bloodstream infections and biofilm-associated diseases [3,4]. HIV-infected patients who are colonized with opportunistic pathogens are at a higher risk for subsequent infections, as oral *Candida* colonization has been identified as a predictor for the occurrence of oral candidiasis, and individuals colonized with MRSA are more likely to develop MRSA infections, usually by the same colonizing strains, than those who are not colonized [5,6]. However, little is known about the frequency of co-colonization by both species in the HIV-infected population.

Earlier work examining 162 HIV-infected patients (median CD4 count 205 cells/mm^3^) in 1999 found a 6% prevalence of nasal MRSA carriage, with ciprofloxacin use and reduced CD4 count being two risk factors for carriage [7]. In Taiwan, the number of HIV-infected patients receiving highly active antiretroviral therapy (HAART) with a favorable CD4 count and suppressed viral load has increased in the recent decade due to the availability of free HAART and implementation of a case management program [8]. Since 1999, the authors have examined the prevalence of and risk factors for oral *Candida* colonization among HIV-infected outpatients at three hospitals during 2009–2010 in three studies [9–11].

In Taiwan, community-associated MRSA (CA-MRSA) emerged around 2002 and has spread to healthcare settings [2,12]. For the epidemiological surveillance of MRSA, HIV-infected outpatients may serve as a sentinel population because of their frequent contact with both hospital and community settings and exposure to antimicrobial agents. Taken together, these epidemiological changes suggested the need for reevaluating the prevalence of and risk factors for MRSA carriage among the contemporary HIV-infected population. Here, the same study population from 2009–2010 [9–11] was enrolled to investigate (1) the prevalence of and risk factors for nasal and oral *S. aureus* and MRSA colonization in order to compare with those for oral *Candida* colonization, and (2) the microbiologic characteristics and clonality of the MRSA colonizing strains. The study results could be helpful for targeted MRSA screening and transmission prevention in this population and expanding our knowledge about the interplay between *S. aureus* and *Candida* species in the oral cavity.

## Material and methods

### Study population and sample collection

This prospective, cross-sectional, multicenter study enrolled HIV-infected outpatients who attended the clinics at China Medical University Hospital, E-Da hospital, and National Cheng Kung University Hospital from October 2009 to February 2010. The hospitals are located in three different counties in Taiwan and are 25–122 miles apart. The Institutional Review Board of each participating hospital approved the study.

After informed consent, the patients were tested for nasal MRSA and oral *Candida* colonization simultaneously. In addition, oropharyngeal culture for *S. aureus* was performed on a subset of these patients. All specimens were obtained using dry sponge swabs (EZ Culturette; Becton Dickinson, Sparks, MD), maintained at room temperature, and transported to the central laboratory at the National Health Research Institutes within 24 h where they were plated on solid medium within 4 h of arrival.

Clinical data collected included demographic characteristics, clinical and laboratory information within 6 months prior to enrollment such as history of incarceration, the latest CD4 cell count and plasma HIV viral load, antibacterial or antifungal treatments for ≥1 day and antiretroviral agents for ≥2 weeks, and history of hospitalization within 1 year prior to enrollment. A suppressed HIV viral load is defined as plasma HIV RNA level <200 copies/mL [13]. Data on oral *Candida* colonization from these patients in the three hospitals have been published separately [9–11] and were merged in this study in order to determine if the risk factors for *S. aureus*, MRSA, and *Candida* colonization differed.

### Microbiological processing

Both the nasal and oral swabs were plated on sheep blood and ChromAgar *S. aureus* plates (BBL Microbiology System, Cockeysville, MD), plus Trypticase soy enrichment broth containing 7.5% NaCl. After overnight incubation at 35ºC, 10 µL of the enrichment broth was subcultured to ChromAgar *S. aureus* and ChromAgar MRSA plates. All colonies suspected to be *S. aureus* were checked by catalase and Gram stain as necessary. All *S. aureus* isolates were confirmed by coagulase/protein A latex agglutination. Semi-quantification of the bacterial load was recorded as 1+ to 4+ based on number of colonies in the streaked four quadrant areas on primary culture plates, and as ‘rare’ if isolates were recovered only from enrichment broth subculture. All *S. aureus* isolates were screened for oxacillin (methicillin) resistance using a cefoxitin disk diffusion test following the protocol of Clinical and Laboratory Standards Institute (CLSI) [14]. Susceptibility to different agents was then determined on all MRSA isolates by broth microdilution following the CLSI guidelines using custom-designed Sensititre plates (Trek Diagnostics, East Grinstead, United Kingdom) [14]. The CLSI breakpoints (MICs in μg/mL) for non-susceptibility to chloramphenicol (>8), ciprofloxacin (>1), clindamycin (>0.5), erythromycin (>0.5), gentamicin (>4), rifampin (>1), tetracycline (>4), co-trimoxazole (>2/38), daptomycin (>1), linezolid (>4), teicoplanin (>8), and vancomycin (>2) were used [14]. All MRSA isolates were subject to SCC*mec* typing and Panton-Valentine leukocidin (PVL) gene detection, as described previously [15]. Clonal relatedness of the MRSA isolates was also investigated by pulsed-field gel electrophoresis (PFGE), and pulsotypes were assigned to clusters of isolates having >80% similarity from the dendrograms [16]. Multilocus sequence typing (MLST) was performed on isolates selected from each pulsotype, and sequence type (ST) was assigned by using the MLST database Web site (www.mlst.net) [17]. Detailed protocols for the culture and identification of *Candida* species have been described previously [9–11].

### Risk factors and statistical analysis

Statistical analyses were performed with the SPSS Statistics for Windows v17.0 (SPSS Inc., Chicago, IL). All categorical variables presented in Table 1 (except the individual antiretroviral agent) were tested for their association with colonization. Variables with a *p*-value of ≤0.10 in univariate analysis were included in multivariate analysis. A *p*-value of <0.05 was considered statistically significant, and all tests were two tailed.

**Table 1.** Characteristics of HIV-infected patients with *S. aureu*s, MRSA, and *Candid*a colonization.

Characteristic (mean ± *SD*) or *n* (%)	All (714) patients
Age, years	38.2 ± 11.7
Age >50 years	107 (15.0)
Gender, male	635 (91.5)
Transmission type	
Heterosexual	193 (27.0)
IDU	87 (12.2)
MSM	419 (58.7)
Comorbidity	
Chronic kidney diseases	2 (0.3)
Diabetic mellitus	20 (2.8)
Hypertension	27 (3.8)
Hospitalization within 1 year	68 (9.5)
Incarceration within 6 months	13 (1.8)
Period of HIV infection, years	5.2 ± 4.0
CD4, cells/mm^3^	472 ± 257
CD4 count <200	93 (13.0)
CD4 count >500	293 (41.0)
HIV viral load, log (copies/mL)^a^	2.3 ± 1.3
HIV viral load <200 copies/mL^a^	481 (67.5)
Medications within 6 months	
Antiretroviral therapy	567 (79.4)
3TC or 3TC/AZT	550 (77.0)
Abacavir	197 (27.6)
AZT or 3TC/AZT	281 (39.4)
NNRTI^b^	263 (36.8)
PI^c^	308 (43.1)
Antibacterials^d^	65 (9.1)
Co-trimoxazole	46 (6.4)
Cephalosporins	13 (1.8)
Anti-tuberculosis regimens^e^	14 (2.0)
Antifungals	
Amphotericin B	3 (0.4)
Fluconazole	12 (1.7)

^a^Data for two patients were not available.

^b^NNRTIs: efavirenz or nevirapine.

^c^PIs: azatanavir/ritonavir, indinavir, or lopinavir/ritonavir.

^d^Antibacterial agents, i.e. clindamycin, fluoroquinolones, macrolides, and penicillin derivatives, each of which was used by fewer than five patients and which was not associated with colonization by either pathogen, were also included.

^e^Of 14 patients, 13, 3, 8, and 11 received ethambutol, isoniazid, pyrazinamide, rifabutin/rifampin, respectively.

HIV, human immunodeficiency virus; *S. aureus, Staphylococcus aureus*; MRSA, methicillin-resistant *S. aureus; SD*, standard deviation; 3TC, lamivudine; AZT, zidovudine; IDU, intravenous drug use; MSM, men having sex with men or bisexual.

## Results

### Study population

A total of 714 patients were studied for nasal *S. aureus* colonization, and a subset of 457 patients were also screened for oral *S. aureus* colonization. The mean age of all patients was 38.2 years (range 18–85 years), and the majority (91.9%) were male. Men who have sex with men (MSM) comprised the largest proportion (58.7%), followed by heterosexual patients (27.0%) and intravenous drug users (IDUs; 12.2%). Thirteen patients, all IDUs, had a history of recent incarceration. At enrollment, most patients (79.4%) were receiving HAART, and the mean CD4 count was 472 cells/mm^3^ (Table 1). The demographic data between the 714 all-patient group and the 457 patient subgroup were not statistically different, except that more patients in the all-patient group had hypertension (*p *= 0.025; data not shown).

### Prevalence of colonization

Among the 457-patient subgroup, oral colonization by *S. aureus*, MRSA, and *Candida* species was found in 86 (18.8%), 14 (3.0%), and 231 (50.6%) patients, respectively, while nasal colonization by *S. aureus* and MRSA was found in 145 (31.7%) and 20 (4.4%) patients, respectively. Oral only, nasal only, both oral and nasal colonization, and colonization at any site by *S. aureus* was found in 23 (5%), 82 (17.9%), 63 (13.8%), and 168 (36.8%) patients, respectively, and by MRSA in 5 (1.1%), 11 (2.4%), 9 (2.0%), and 25 (5.5%) patients, respectively. The added yield for MRSA carriage from oral screening was 25.0%. Both oral *S. aureus* and MRSA colonization was positively associated with their nasal colonization (*p *< 0.001 for both). The all-patient group had a similar prevalence of nasal colonization by *S. aureus* (228; 31.9%) and MRSA (28; 3.9%), among which 85.5% (195/228) and 85.7% (24/28) had ≥1+ growth, including 71.1% (162/228) and 71.4% (20/28), respectively, having 2+ or more growth on primary culture plates. In contrast, all *S. aureus* and MRSA from the oral cavity were recovered in a rare amount, since they were detected from enrichment subculture only. The overall oral *Candida* colonization was 52.4%, with *C. albicans* being the most common species identified (73.8%). Of the 457-patient subgroup, 9.2 and 1.5% of patients had *Candida* co-colonization with *S. aureus* and MRSA, respectively. Oral *Candida* (and *C. albicans*; data not shown) colonization was not correlated with either oral or nasal colonization by *S. aureus* or MRSA (Table 2).

**Table 2.** Prevalence of colonization by organisms and sites among 457 HIV-infected outpatients.

	Oral *Candida* colonization		*p*
	Positive, *n* = 231	Negative, *n* = 226	
Oral colonization by *S. aureus*			
Positive, *n* = 86	42 (9.2)	44 (9.6)	0.725
Negative, *n* = 371	189 (41.4)	182 (39.8)	
Oral colonization by MRSA			
Positive, *n* = 14	7 (1.5)	7 (1.5)	0.967
Negative, *n* = 443	224 (49.0)	219 (47.9)	
Nasal colonization by *S. aureus*			
Positive, *n* = 145	76 (16.6)	69 (15.1)	0.586
Negative, *n* = 312	155 (33.9)	157 (34.4)	
Nasal colonization by MRSA			
Positive, *n* = 20	13 (2.8)	7 (1.5)	0.186
Negative, *n* = 437	218 (47.7)	219 (47.9)	

Data are shown as *n* (%).

Patients who were recently incarcerated had a higher risk for MRSA carriage at any site than those who had not been incarcerated (23.1% [3/13] vs. 4.3% [30/701]; *p *= 0.030). The prevalence of oral *S. aureus*, MRSA, and *Candida* colonization was lower among patients with a suppressed viral load than among patients with a higher viral load (≥200 copies/mL; 15.8% vs. 24.7%, *p *= 0.022; 1.7% vs. 5.7%, *p *= 0.024; and 46.5% vs. 57.6%, *p *= 0.024, respectively).

### Microbiologic characteristics and clonality of MRSA

**Figure 1.**
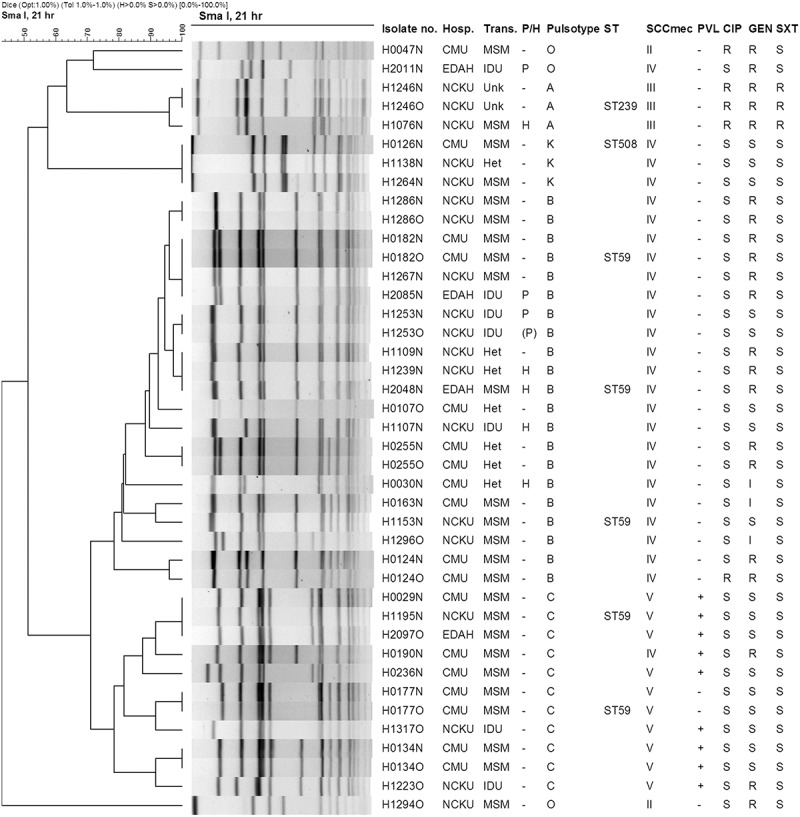
PFGE dendrogram with molecular characterization of nasal and oral MRSA isolates from HIV-infected patients. PFGE cluster was assigned to isolates having ≥80% similarity from the dendrograms. Isolate number indicates the strain number, in which the last letter N and O represent nasal and oral isolates, respectively. Isolates with an identical strain number irrespective of N or O were from the same patient. CIP, ciprofloxacin; Gen, gentamicin; H, hospitalizations; Het, heterosexual; HIV, human immunodeficiency virus; I, intermediate; IDU, intravenous drug user; MRSA, methicillin-resistant *S. aureus*; MSM, men who have sex with men; P, in jails or prisons; FFGE, pulsed-field gel electrophoresis; PVL, Panton-Valentine leukocidin; R, resistant; S, susceptible; ST, sequence type determined by multi-locus sequence typing; SXT, trimethoprim/sulfamethoxazole (co-trimoxazole); Trans, transmission route of HIV in indicated patient.

A total of 41 MRSA isolates were recovered from 33 patients, including eight pairs of nasal and oral isolates (each pair from the same patient) and an additional 19 nasal and 6 oral isolates from different patients. Based on PFGE, the majority of the isolates belonged to two main clusters: pulsotype B (21 isolates, 51.2%; 16 patients, 48.5%) and pulsotype C (11 isolates, 26.8%; 9 patients, 27.3%). Three isolates (7.3%) each belonged to pulsotypes A (from two patients) and K (from three patients), and the remaining three isolates had PFGE patterns distinct from pulsotypes A–C and K (Figure 1). The nasal and oral isolates from the same patient all had indistinguishable PFGE patterns.

Both pulsotypes B and C are associated with ST59 and contained genetically identical or closely related strains from the three hospitals. Pulsotype B isolates were PVL-negative but carried SCC*mec* IV and were recovered from patients who were MSM, heterosexual, IDUs, or recently incarcerated. Pulsotype C isolates were mostly PVL-positive, carried SCC*mec* V_T_, and were recovered from patients who were MSM or IDUs. Pulsotype A isolates are ST239 and carried SCC*mec* III, while pulsotype K isolates are ST508 and carried SCC*mec* IV.

Rates of non-susceptibility to chloramphenicol, ciprofloxacin, clindamycin, erythromycin, gentamicin, rifampin, tetracycline, and co-trimoxazole were 63.4%, 9.8%, 82.9%, 85.4%, 58.5%, 2.4%, 51.2%, and 7.3% (three isolates), respectively, while all isolates were susceptible to daptomycin, linezolid, teicoplanin, and vancomycin. The three co-trimoxazole-resistant isolates all carrying SCC*mec* III were recovered from two patients, one of whom was recently hospitalized and receiving co-trimoxazole prophylaxis.

### Risk factors for colonization

Risk factors for colonization examined by multivariate analysis are presented in Table 3. A suppressed HIV viral load protected against oral colonization by *S. aureus* and MRSA but not against their nasal colonization. Recent antibacterial use was associated with a lower risk of both nasal and oral *S. aureus* colonization, and, in particular, recent co-trimoxazole use was negatively associated with nasal *S. aureus* colonization. Older age (>50 years) and IDU were another two independent protective factors for nasal *S. aureus* colonization. Recent incarceration was the only risk factor identified for nasal MRSA colonization. For oral *Candida* colonization, a low HIV viral load remained an independent protective factor, while recent hospitalization, recent tuberculosis (TB) with anti-TB regimens, older age, and IDU increased risk for colonization.

**Table 3.** Summary of predictors for *S. aureu*s, MRSA, and *Candid*a colonization in the nasal and oral cavity of HIV-infected outpatients.

Status	Predictors	Characteristic	Of 457 subjects^a^		Of all (714) subjects^b^	
			*p*	OR (95% CI)	*p*	OR (95% CI)
Oral *S. aureus* colonization	HIV VL <200	Protective	0.010	0.53 (0.33–0.86)	NA	–
	Antibacterial agent use	Protective	0.045	0.22 (0.05–0.97)	NA	–
Oral MRSA colonization	HIV VL <200	Protective	0.026	0.28 (0.09–0.86)	NA	–
Nasal *S. aureus* colonization	Age >50 years	Protective	NS	–	0.028	0.58 (0.36–0.94)
	Antibacterial agent use	Protective	0.028	0.34 (0.13–0.89)	NS	–
	Co-trimoxazole use	Protective	NS	–	0.028	0.39 (0.17–0.91)
	Intravenous drug user	Protective	0.001	0.26 (0.12–0.59)	0.001	0.36 (0.20–0.65)
Nasal MRSA colonization	Incarceration	Risk	NS	–	0.002	8.11 (2.10–30.31)
MRSA colonization at any site	None		–	–	NA	–
Oral *Candida* colonization	Age >50 years	Risk	0.001	2.68 (1.49–4.79)	0.007	1.85 (1.18–2.89)
	HIV VL <200	Protective	0.007	0.57 (0.38–0.85)	0.031	0.60 (0.40–0.97)
	Hospitalization	Risk	0.040	2.27 (1.04–4.98)	0.019	2.07 (1.12–3.81)
	Tuberculosis	Risk	0.059	7.58 (0.93–61.91)	0.028	9.96 (1.28–77.79)
	Intravenous drug use	Risk	NS	–	0.002	2.20 (1.35–3.59)

^a^Excluding the 257 patients not tested for oral *S. aureus* colonization.

^b^All 714 patients were tested for nasal *S. aureus* colonization.

CI, confidence interval; MRSA, methicillin-resistant *S. aureus*; NA, not applicable; NS, not significant; OR, odds ratio; VL, plasma viral load (copies/mL).

## Discussion

Although colonization by either MRSA or *Candida* species among HIV-infected patients has been investigated, this study differed from previous investigations by examining colonization by both species in both the nasal and oral cavities simultaneously in the contemporary HIV-infected population. This study design enabled risk factors to be compared between colonizing organisms and between sites of colonization. Furthermore, a HIV-infected outpatient population was enrolled with a mean CD4 count of 472 cells/mm^3^, among whom a substantial portion were on HAART (79.4%), with a suppressed viral load (67.5%), and a CD4 count of ≥500 cells/mm^3^ (41.0%). In general, the normal CD4 count in healthy adults ranges from 500 to 1,500 cells/mm^3^, and a viral load of ≥200 copies/mL is defined as virologic failure for HIV-infected patients receiving HAART [13,18]. It appeared that most patients enrolled in the present study had their HIV under adequate control and had achieved favorable immunity in terms of these laboratory criteria.

Compared to studies examining MRSA carriage among HIV-infected patients conducted in other parts of world during the past decade, the mean CD4 count of patients in the present study (472 cells/mm^3^) was comparable to those (416–599 cells/mm^3^) reported elsewhere [19,20] but higher than the 205 cells/mm^3^ found in an earlier study by the authors in 1999 [7]. The nasal *S. aureus* carriage rate (31.9%) found in patients in the present study was also similar to those (26.7–3.6%) of other contemporary HIV-infected patients [19,21]. The nasal and oral MRSA carriage rates (3.9–4.3 and 3.0%) found in this study were within the ranges reported from recent parallel HIV studies (i.e. 0–17.3 and 0–8%, respectively) by other study groups [19,20,22].

In addition, higher rates of nasal *S. aureus* and MRSA colonization (31.9% and 3.9–4.3%) than oral colonization (18.8 and 3.0%) have been observed in other HIV-infected populations [20,21]. The finding of higher bacterial loads in the nasal than in the oral cavity here was consistent with a recent microbiota analysis showing that Staphylococcaceae constituted 55% of nasal commensals but was comparatively uncommon in the oral cavity [23]. Despite this, several oral-positive/nasal-negative *S. aureus* and MRSA carriers were identified, resulting in an overall MRSA carriage rate of 5.5%. The added yield for MRSA carriage from oral screening was 25%, similar to the average added yield (17.5%) from screening by throat culture summarized in a meta-analysis [20]. Hence, it should be borne in mind that nasal-only screening would underestimate the MRSA carriage rate.

Although CA-MRSA infection has emerged in Taiwan since 2002, the nasal MRSA carriage rate here (3.9%) did not increase over the years compared with the rate (6%) found in the authors’ 1999 study, and was similar to the rate (3.8%) reported among 3,098 healthy adults in Taiwan in 2007 [7,24]. Different from the United States where the prevalent CA-MRSA clone, USA300 (ST8/SCC*mec* IV/*pvl*-positive), which has also been found to be the leading strain colonizing HIV-infected patients [20], the CA-MRSA isolates in Taiwan can be grouped into two distinct clones: the Asian-Pacific clone (ST59/pulsotype B/SCC*mec* IV/PVL-negative) and the virulent Taiwan clone (ST59/pulsotype C/SCC*mec* V_T_/PVL-positive) [2]. Both are susceptible to co-trimoxazole. In the present study, 48.5% of patients were colonized by pulsotype B isolates with genotypes characteristic of the Asian-Pacific clone, and 27.3% were colonized by pulsotype C isolates having the molecular characteristics of the virulent Taiwan clone. Two Taiwanese studies investigating nasal MRSA carriage among healthy adults and children also revealed similar findings [24,25]. These observations indicated that among HIV-infected outpatients comprising a high proportion on HAART, the MRSA carriage rate appeared to be similar to that of the general population, and most of the colonizing isolates belonged to typical CA-MRSA strains circulating locally. However, a few patients were also identified who were colonized by ST239/SCC*mec* III isolates, the typical healthcare-associated MRSA strains in Taiwan, likely due to recent hospitalization [2].

Three patients were also found who were colonized with ST508/SCC*mec* IV MRSA. A previous study in Taiwan found ST508 to be the most common nasal carriage clone of methicillin-susceptible *S. aureus* (MSSA), but it was rarely detected in the MSSA infection strains [26]. In addition, although there have been a few reports of ST508 *S. aureus*, most are MSSA and rarely MRSA [27]. ST508 is a single locus variant of ST45 belonging to clonal complex 45 (CC45). Since ST45 MRSA has emerged in Taiwan in recent years [28], further studies to determine the association of CC45 MRSA and MSSA might shed light on their evolution.

Multivariate analysis revealed that a suppressed HIV viral load protected against oral *Candida* colonization, consistent with a previous study demonstrating that high HIV viral load was a significant predictor for oral *Candida* colonization [29]. Further, it was found that a suppressed viral load protected against oral colonization by *S. aureus* and MRSA as well, which has not been reported previously. It has been shown that the phagocytic activity of monocytes and neutrophils can be directly suppressed by HIV but restored by lowering HIV load [30]. Such direct and other indirect effects exerted by HIV might alter oral mucosal immunity that promotes colonization and infection by commensal or pathogenic organisms [29,30]. In contrast, effective management of HIV infection by HAART, reflected by a decreased viral load, might restore oral immunity and subsequently decrease microbial colonization and infection, as observed in this study [29,30]. Notably, although a CD4 count <200 cells/mm^3^ was identified as a risk factor for oral *Candida* colonization in the authors’ previous work in which the viral load was not included for analysis [11], the association between a low CD4 count and oral colonization by either *S. aureus* or *Candida* was not demonstrated here. This is in agreement with clinical findings that plasma HIV-RNA levels, regardless of CD4 count, was the only correlate of oral *Candida* colonization, and oral candidiasis usually resolved after initiation of HAART, even though the CD4 count remained low [29]. These implied that improvements in the immune function after HAART are not reflected solely by an increase of CD4 count, which might lag behind other immunological parameters [29]. On the other hand, a suppressed viral load did not protect against nasal *S. aureus* and MRSA colonization, as found in most studies [20]. Together with the observation of more abundance of Staphylococcaceae in the nasal cavity, the mucosal immunity against *S. aureus* colonization appears to differ between the nasal and oral microenvironments.

Synergic interaction between *Candida* species (and *C. albicans*) and *S. aureus* in oral mucosal colonization was not demonstrated here and is rarely mentioned in clinical studies. *C. albicans* usually appear as non-invasive oral commensals, which, under conditions of immune dysfunction, can rapidly transition to pathogens through morphological switch from the rounded yeast to the invasive hyphal form, causing local or systemic diseases [31]. Furthermore, *C. albicans* can facilitate the invasion of *S. aureus* across host mucosal barriers, leading to systemic infection, resulting from the high affinity of *S. aureus* to the invasive hyphal elements (but not to the yeast form) of *C. albicans* and the propensity of *C. albicans* to adhere to and penetrate tissue via its invasive hyphae [31]. Lack of synergy found here might be explained by the fact that most patients enrolled have achieved favorable immunity, which restricted the *Candida* yeast-to-hyphae transition and subsequent bounding between the *Candida* hyphae and *S. aureus*. Though further studies are needed, it seems that under restored immunity, *Candida* spp. does not augment *S. aureus* colonization in the oral mucosa, which reduces the possibility of co-colonization and further progression to systemic diseases and may in theory be another benefit of HAART.

Recent antibacterial agent use, particularly co-trimoxazole, was another protective factor against nasal and oral *S. aureus* colonization. This is consistent with the fact that co-trimoxazole remained active against most community-associated *S. aureus* isolates, including CA-MRSA in Taiwan found in the present study and elsewhere [24,26]. The positive association of prior incarceration with nasal MRSA colonization has also been documented in several studies [20]. This could be explained by the fact that the nostrils are more susceptible to *S. aureus* colonization and more frequently in contact with the environmental microbes compared to the oral cavity, and prisons could be a reservoir of CA-MRSA due to overcrowding and unsanitary conditions [20,23]. Hence, adherence to general hygiene and infection-control practices is emphasized in order to reduce MRSA transmission in crowded institutions.

Recent hospitalization, tuberculosis, older age, and IDU were risk factors for oral *Candida* colonization. Although the association of tuberculosis with colonization has been demonstrated in two other studies [32,33], it is not known whether broad-spectrum antibacterial activity exerted by anti-tuberculosis regimens played a role, or whether tuberculosis served as a confounder reflecting impaired immunity or other unmeasured risk factors for colonization, which merits further studies. Older age and IDU increased the risk of oral *Candida* colonization, which is similar to previous studies showing that both groups had higher rates of oral *Candida* colonization [34,35]. Several factors, such as denture use, oral hygiene, smoking, and access to healthcare, should be evaluated in order to delineate the impact of age and IDU on oral *Candida* colonization.

This study has a few limitations. First, a HIV-uninfected population was not enrolled. However, a separate large-scale surveillance for MRSA carriage among 3,098 healthy adults with a similar average age (mean 39 years) and rate of hospitalization within 1 year (5.3%) that was conducted around the same study period could serve as a comparable reference group [24]. Second, patients in the present study were not followed up for subsequent infections. Therefore, the clinical impact of MRSA colonization by different clones, and that of *Candia* and *S. aureus* co-colonization, was not clear.

In conclusion, the finding of a lower rate and bacterial burden of *S. aureus* oral carriage compared with those of *S. aureus* nasal carriage and rate of *Candida* oral carriage suggested that the mucosal immunity against colonization might differ in terms of microbes and sites. Among HIV-infected outpatients, most of whom were receiving HAART, synergic interaction between *S. aureus* and *Candida* spp. in the oral mucosa was not demonstrated, and a suppressed viral load protected against *S. aureus*, MRSA, and *Candida* oral colonization, both of which might be benefits of HAART. Patients with recent incarceration were at risk for nasal MRSA colonization and hence should be carefully screened if clinically indicated.
